# Five-Compressions Protocol as a Valid Myotonometric Method to Assess the Stiffness of the Lower Limbs: A Brief Report

**DOI:** 10.3390/ijerph192114425

**Published:** 2022-11-03

**Authors:** Alfredo Bravo-Sánchez, Pablo Abián, Jorge Sánchez-Infante, María Ramírez-delaCruz, Paula Esteban-García, Fernando Jiménez, Javier Abián-Vicén

**Affiliations:** 1 Faculty of Health Sciences, Universidad Francisco de Vitoria, 28223 Pozuelo de Alarcón, Spain; 2 Faculty of Humanities and Social Sciences, Comillas Pontifical University, C. Alberto Aguilera 23, 28049 Madrid, Spain; 3 Hospital Universitario de Toledo, Castilla La Mancha, 45007 Toledo, Spain; 4 Performance and Sport Rehabilitation Laboratory (DEPORSALUD), Faculty of Sports Sciences, University of Castilla-La Mancha, Avda. Carlos III s/n, 45071 Toledo, Spain

**Keywords:** MyotonPRO, mechanical properties, lower limbs, muscle, tendon

## Abstract

The objective of this study was to evaluate the validity of a short assessment MyotonPRO protocol to measure the stiffness of the superficial muscles and tendons of the lower limbs. The stiffness of the dominant lower limb vastus lateralis (VL), rectus femoris (RF) and patellar tendon (PT) was evaluated in 52 healthy participants (26.9 ± 3.4 years) with two MyotonPRO protocols: the standard protocol (10 mechanical taps) and the short protocol (five mechanical taps). The myotonometry was performed at the midpoint of the length from the upper pole of the patella to the greater trochanter for the VL, and to the anterior superior iliac spine for the RF. The PT was evaluated 1 cm caudal from the inferior pole of the patella. Pearson’s correlation coefficients were calculated to determine the relationships between protocols. The validity of the short protocol was evaluated with Student’s *t*-test. High positive correlations were observed between the short and standard protocols in the stiffness of the VL (r = 0.959; *p* < 0.001), the RF (r = 0.967; *p* < 0.001) and the PT (r = 0.953; *p* < 0.001) and no differences were found between both protocols in the stiffness assessment of the VL, RF and PT (*p* > 0.05). Therefore, the five-compressions protocol is a valid protocol for the assessment of lower limb mechanical properties.

## 1. Introduction

Stiffness is one of the most studied mechanical properties of muscles and tendons because of its positive relation with the rate of force development and performance in fast stretch shortening cycle activity [1]. Elastography ultrasound is widely used to quantify the mechanical properties of tissues, and has been considered to be the gold standard for quantifying the Young’s modulus (stiffness) of the tissue [2,3]. The high operator dependence of elastography and its considerable economic cost promoted the development of new tools to assess myotendinous mechanical properties. The MyotonPRO is a valid handheld device that measures muscle and tendon mechanical properties non-invasively and objectively. The myotonometer applies repetitive short (15 ms) and low-intensity (0.58 N) mechanical impulses on the skin overlaying the muscle, and the tissue response is analysed by the software of the device to obtain the data on oscillation frequency, stiffness and logarithmic decrement [4,5].

Several studies have shown the feasibility of using the MyotonPRO device to monitor the mechanical properties of skeletal muscles in athletes, healthy young and older adults, and individuals with some pathology [6,7,8]. These studies analysed muscle mechanical properties using the standard protocol with ten brief mechanical stimuli, and it demonstrated moderate to excellent intra- and inter-evaluator reliability (intra-class correlation coefficients (ICC) ranging from 0.62 to 0.99) [5,6,9]. Finally, the MyotonPRO has also been shown to be a useful tool for finding differences in stiffness between both muscle groups and pre-training and post-training moments, and even as a valid diagnostic method to describe the presence of myofascial trigger points [10,11].

Reducing the time duration of the standard measurement protocol would facilitate the application of the MyotonPRO within more complex assessment processes and help to increase its efficiency, as overall test time would be lower. Some authors have employed shorter protocols for measuring skin mechanical properties, and have obtained good reliability results [12], although the validity of this new protocol has not been reported. Therefore, the purpose of the present study was to evaluate the validity of a short MyotonPRO protocol to measure the stiffness of superficial muscles and tendons of the lower limbs.

## 2. Materials and Methods

### 2.1. Sample

A total of 52 physically active people volunteered to participate in this investigation ((mean ± standard deviation) age: 26.9 ± 3.4 years, height: 173.8 ± 12.7 cm, body weight: 72.9 ± 11.6 kg). The sample size was previously calculated based on Schneebeli et al. [13], who used MyotonPRO to measure the effects of muscle contraction intensity on Achilles tendon mechanical properties. The minimal number of subjects required to attain a power of 0.9 and a bilateral alpha level of 0.05 was calculated to be 17. The inclusion criteria were that participants were not to be afflicted with any injury or pain that would prevent them from doing their usual sports practice. All participants were informed regarding the purpose and procedures of the investigation and signed an informed consent form before the commencement of the study.

### 2.2. Design and Procedure

Two protocols were employed to assess the mechanical properties of three dominant lower limb regions: the Vastus Lateralis (VL), the Rectus Femoris (RF) and the Patellar Tendon (PT), using a hand-held myotonometer Myoton^®^ Pro (Myoton AS, Tallinn, Estonia). All the measurements were taken by the same expert operator (ABS). During the assessment with the myotonometer, the testing probe was placed perpendicular to the skin at the reference point. The MyotonPRO was set up with a multiscan sequence (1 s apart) of a short duration (15 ms) impulse involving minimal mechanical force (0.4 N) and a light precompression force (0.18 N). The difference between protocols consisted of the number of taps or impulses that composed the multiscan sequence. The first protocol (standard protocol) consisted of 10 mechanical taps whereas the second protocol consisted of 5 mechanical taps (short protocol). The mean value of the data from the 10 and 5 compressions protocols was used to describe the oscillation frequency, logarithmic decrement and stiffness of the examined muscles and tendon. 

The anatomical locations of the measurement points and participants’ positions were standardised for all subjects. MyotonPRO exams were performed at: the midpoint from the upper pole of the patella to the greater trochanter for the VL; at the midpoint from the upper pole of the patella to the anterior superior iliac spine for RF and at 1 cm caudal from the inferior pole of the patella for PT.

The participants were evaluated twice in random order, using different protocols each time (standard and short protocol), to be able to compare the validity of the short protocol. All the measurements were performed in a relaxed condition, and the participant was requested to not contract their muscles. For the assessment of the VL and RF the participants were laid in a supine position with the knee full extended and, for the PT assessment the participants were laid in a supine position with 30° of knee flexion, using a foam pad to support the position [6].

### 2.3. Data Analysis

Data analysis was performed using SPSS v 28.0 (SPSS Inc., Chicago, IL, USA). All data were expressed as a mean with standard deviation. The data were tested for normality with the Shapiro-Wilk’s test. Given the assumption of normality (all variables *p* > 0.05), the validity of the short protocol was evaluated with Student’s *t*-test and the Bland-Altman plot. Pearson correlation coefficients were used to examine the relations between the standard and short protocols. A paired sample Student’s *t*-test was performed to analyse the inter-region differences in stiffness values measured with both protocols. The inter-region differences in stiffness (VL vs. RF; VL vs. PT; RF vs. PT) were calculated for each protocol result as follows:

Difference (N/m) = Stiffness region A − Stiffness region B.(1)

A paired sample Student’s *t*-test was performed to identify the change in the mean of these differences between protocols (standard protocol vs. short protocol). The effect size was interpreted using Cohen’s d [14]: an effect size less than 0.2 was considered small; an effect size of about 0.5 was considered medium; and a large effect size was considered when the result was greater than 0.8. For all statistical analyses, *p* < 0.05 was accepted as the level of significance.

## 3. Results

### 3.1. Validity Results

**Figure 1:**
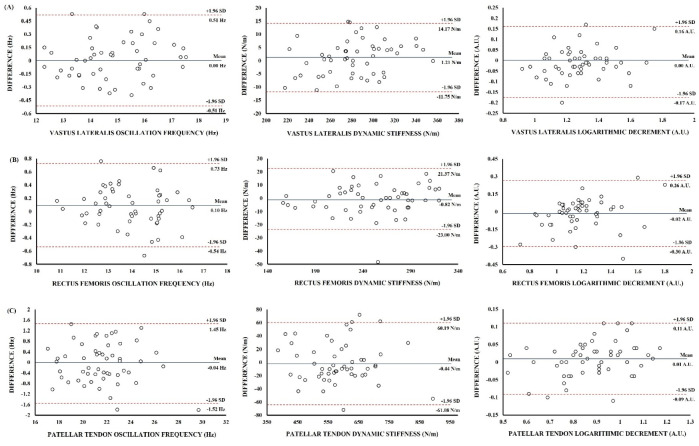
Bland-Altman plot showing the limits of agreement between short and standard protocol measurement: (**A**) Vastus Lateralis; (**B**) Rectus Femoris; (**C**) Patellar Tendon. The centre line represents the mean differences between the two protocols, and the other two lines represent two SDs.

Student’s *t*-test did not show significant differences between protocols (standard protocol vs. short protocol) for any mechanical properties (*p* > 0.05) (Table 1). The Bland-Altman plot showed no systematic bias, as the data points were distributed equally below and above the mean for all mechanical properties (Figure 1).

**Figure 2:**
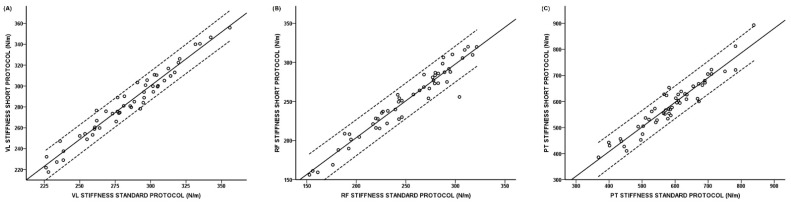
Scatter-plot with regression line of the relationship between short protocol and standard protocol: (**A**) Vastus Lateralis (VL); (**B**) Rectus Femoris (RF); (**C**) Patellar Tendon (PT).

Significant positive correlations were revealed between the standard and short protocol for VL oscillation frequency (r = 0.984; *p* < 0.001), VL stiffness (r = 0.959; *p* < 0.001; Figure 2), VL logarithmic decrement (r = 0.906; *p* < 0.001), RF oscillation frequency (r = 0.973; *p* < 0.001), RF stiffness (r = 0.967; *p* < 0.001; Figure 2), RF logarithmic decrement (r = 0.784; *p* < 0.001), PT oscillation frequency (r = 0.952; *p* < 0.001), PT stiffness (r = 0.953; *p* < 0.001; Figure 2) and PT logarithmic decrement (r = 0.947; *p* < 0.001).

### 3.2. Inter-Region Differences Results

The results of inter-region analysis showed that the PT experienced greater stiffness than the VL (d = 4.0; *p* < 0.001) and the RF (d = 4.3; *p* < 0.001) in the short protocol, and the PT stiffness was also significantly higher than the VL stiffness (d = 4.1; *p* < 0.001) and the RF stiffness (d = 4.3; *p* < 0.001) in the standard protocol (Table 1). In addition, greater stiffness values were reported for the VL than the RF in both protocols: short protocol (d = 0.9; *p* < 0.001) and standard protocol (d = 0.9; *p* < 0.001) (Table 1).

### 3.3. Inter-Protocol Differences Results

**Figure 3:**
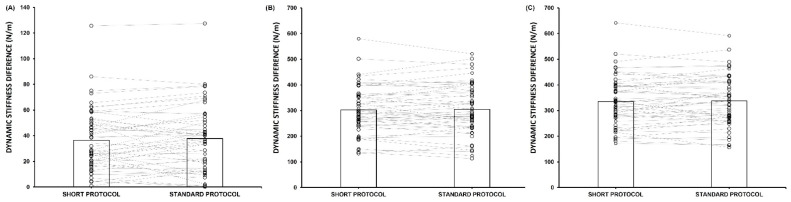
Comparison of inter-region stiffness differences measured with the short and standard MyotonPRO protocols: (**A**) Vastus Lateralis vs. Rectus Femoris; (**B**) Patellar Tendon vs. Vastus Lateralis; (**C**) Patellar Tendon vs. Rectus Femoris.

Figure 3 shows the inter-protocol differences in mean difference of region comparisons. No differences were found between the standard and short protocol (*p* > 0.05).

## 4. Discussion

The current study aimed at validating a new measurement protocol of the MyotonPRO application for assessing myotendinous mechanical properties. Reducing the measurement time of the MyotonPRO application would improve efficiency in research studies and increase the possibilities of the MyotonPRO application for the assessment of myotendinous mechanical properties in different research environments. The short measurement protocol was applied previously in the study of the Achilles tendon mechanical properties with different levels of Triceps Surae muscle contraction [13] and during the exam of the Achilles tendon stiffness in different ankle positions [15], good reliability results were obtained, although no validity study was done. Based on our results, we found that the short protocol is a valid protocol for assessing the mechanical properties of the VL, RF and PT, therefore the measurement time may be reduced from ~15 s to ~7 s. This time reduction would allow the application of the MyotonPRO in studies where a short period of time is available to analyse myotendinous mechanical properties where only shear wave elastography [16] has been applicable to date. Therefore, we suggest the utilisation of the short protocol by researchers for the evaluation of lower limb myotendinous mechanical properties.

Several studies showed potential differences in MyotonPRO parameters between the evaluated regions (inter-muscle differences) and sex-related differences [17,18], and the free tendon region and the myotendinous junction [19]. In our research, we found higher values of stiffness in those regions with greater collagen composition (PT) than regions with lower collagen composition (VL and RF), coinciding with previous studies [18]. The short protocol was designed to measure the myotendinous mechanical properties and to discriminate between the different regions (muscles and tendons) in lower limbs in the same way that the standard measurement has done to date, achieving a reduction in the time assessment of the MyotonPRO application. As reported with the standard protocol, short protocol results showed greater stiffness in the PT than in the VL and RF. To validate the use of the short MyotonPRO protocol in daily clinical practice and in different research environments, we compared the magnitude of inter-region differences between both protocols. Considering that the differences in myotonometric parameters between regions measured with the standard and short protocols were equal after inter-protocol comparison, the short protocol showed similar discriminant ability of myotendinous stiffness to the standard protocol.

Finally, we evaluated the relationship between the two measurement protocols using Pearson’s r statistic, which allowed us to establish a linear relationship between both protocols. The results obtained in our research have shown a significant high positive relationship between both measurement protocols for all the analysed variables. According with Schober et al. [20], r values close to 1 supposed a high reliability between measurement protocols and are also related to a low unexplained variability coefficient. Therefore, after our correlation analysis we can conclude that the short protocol is a reliable method for assessing myotendinous stiffness.

### Limitations

This work has some limitations. MyotonPRO measurements could be conditioned by the thickness of the adipose tissue under the probe, so our results should be corroborated for other body regions with thicker adipose tissue. In addition, only a specific sample of healthy and young people was included in this study, so it would be necessary to apply the study in a population sample with different characteristics. Finally, all the exams were done in a relaxed condition, without muscle contraction, so new research is required in dynamic conditions, during muscle contraction.

## 5. Conclusions

In conclusion, our study demonstrated that the five-compressions protocol (short protocol) is valid for the assessment of lower limb myotendinous mechanical properties. Therefore, the time for myotonometer assessment could be reduced in future research works and daily clinical practice.

## Figures and Tables

**Table 1:** Mechanical property results of measured muscles and tendon of healthy volunteers (mean ± standard deviation).

	FR (Hz)	D (A.U.)	Stiffness (N∙m^−1^)
Vastus Lateralis			
Standard Protocol	14.85 ± 1.38 ^†^	1.28 ± 0.18 ^†^	282.86 ± 30.79 ^†^
Short Protocol	14.80 ± 1.44 *	1.29 ± 0.19 *	283.54 ± 32.43 *
Δ [IC 95%]	0.05 ± 0.26 [−0.12 to 0.02]	0.01 ± 0.08 [−0.01 to 0.04]	0.68 ± 9.23 [−1.89 to 3.24]
Rectus Femoris			
Standard Protocol	13.85 ± 1.36 ^†‡^	1.18 ± 0.22 ^†‡^	249.89 ± 44.79 ^†‡^
Short Protocol	13.94 ± 1.35 *^#^	1.17 ± 0.20 *^#^	250.76 ± 43.62 *^#^
Δ [IC 95%]	0.09 ± 0.31 [−0.01 to 0.17]	0.01 ± 0.14 [−0.05 to 0.03]	0.87 ± 11.42 [−2.31 to 4.05]
Patellar tendon			
Standard Protocol	21.67 ± 2.48	0.87 ± 0.15	588.51 ± 101.95
Short Protocol	21.63 ± 2.48	0.88 ± 0.15	586.15 ± 101.17
Δ [IC 95%]	0.05 ± 0.76 [−0.26 to 0.16]	0.01 ± 0.05 [−0.01 to 0.02]	2.36 ± 31.09 [−11.02 to 6.29]

FR: Frequency; D: Logarithmic Decrement; ^†^ differences from Patellar Tendon in Standard Protocol; ^‡^ differences from Vastus Lateralis in Standard Protocol; * differences from Patellar Tendon in Short Protocol; ^#^ differences from Vastus Lateralis in Short Protocol; significance *p* < 0.05.

## Data Availability

The data presented in this study are available on request from the corresponding author. The data are not publicly available due to restrictions of the subjects’ agreement.
